# Necrotizing Soft Tissue Infection Occurring after Exposure to *Mycobacterium marinum*

**DOI:** 10.1155/2014/702613

**Published:** 2014-11-19

**Authors:** Shivani S. Patel, M. Lance Tavana, M. Sean Boger, Soe Soe Win, Bassam H. Rimawi

**Affiliations:** ^1^Division of Infectious Diseases, Department of Internal Medicine and Dermatology, Medical University of South Carolina, 96 Jonathan Lucas Street, Charleston, SC 29425, USA; ^2^Division of Plastic Surgery, Department of General Surgery, Medical University of South Carolina, 96 Jonathan Lucas Street, Charleston, SC 29425, USA; ^3^Division of Infectious Diseases, Department of Medicine, Medical University of South Carolina, 96 Jonathan Lucas Street, Charleston, SC 29425, USA; ^4^Division of Maternal Fetal Medicine, Department of Gynecology and Obstetrics, School of Medicine, Emory University, 550 Peachtree Street, Atlanta, GA 30308, USA

## Abstract

Cutaneous infections caused by *Mycobacterium marinum* have been attributed to aquarium or fish exposure after a break in the skin barrier. In most instances, the upper limbs and fingers account for a majority of the infection sites. While previous cases of necrotizing soft tissue infections related to *M. marinum* have been documented, the importance of our presenting case is to illustrate the aggressive nature of *M. marinum* resulting in a persistent necrotizing soft tissue infection of a finger that required multiple aggressive wound debridements, followed by an amputation of the affected extremity, in order to hasten recovery.

## 1. Introduction

Cutaneous infections caused by* Mycobacterium marinum* have steadily increased, with over 850 additional cases reported in humans [1]. These cutaneous infections are generally attributed to aquarium or fish exposure after a break in the skin barrier [2]. In most reported cases, the upper limbs and fingers account for most of these infection sites [3, 4]. Treatment should be tailored to prevent progression to deeper infections and hasten recovery [4].

Optimal treatment usually involves appropriate drug therapy supplemented by surgical intervention for medical failure [4]. No prior case reports describe the aggressive nature of* M. marinum* resulting in a persistent necrotizing soft tissue infection of an affected extremity, ultimately requiring multiple aggressive wound debridements, followed by an amputation of the affected extremity, in order to hasten recovery.

## 2. Case Presentation

**Figure 1:**
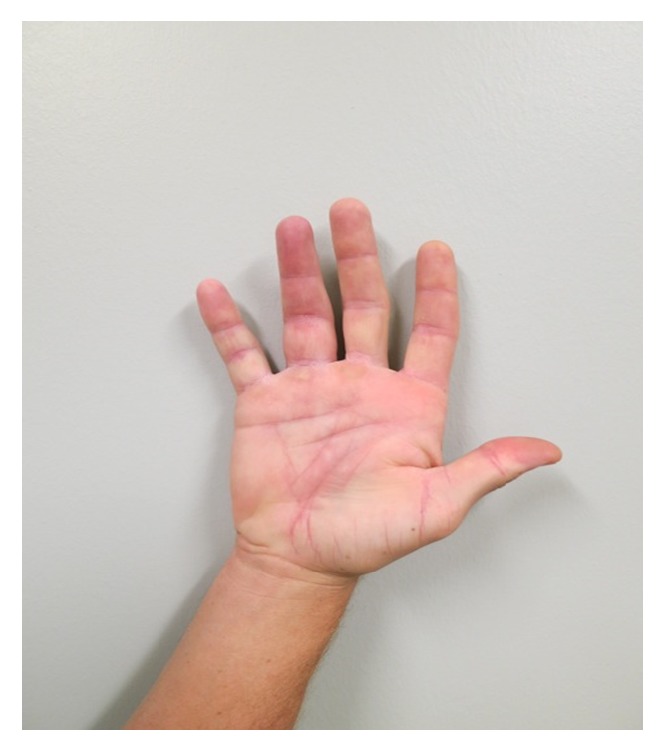
Initial presentation of injury. Three weeks after a barb fish spine penetrated the volar aspect of the proximal portion of his ring finger on his right hand. The injury occurred while fishing along one of the Charleston charters in August. He complained of a gradual increase in pain and swelling of the finger. On physical examination, entry and exit points were noted on the affected finger. Marked swelling was present; however, no bruising, cellulitis, discharge, erythema, or induration was noted. He was able to extend and flex the finger.

A 37-year-old Caucasian male fisherman, with no significant past medical history, presented to the emergency department three weeks after a barb fish spine penetrated the volar aspect of the proximal portion of his right ring finger (Figure 1). The injury occurred while fishing along one of the Charleston charters in August. He complained of a gradual increase in pain and swelling of the finger. On physical examination, entry and exit points were noted on the affected finger. Marked swelling was present; however, no bruising, cellulitis, discharge, erythema, or induration was noted. He was able to extend and flex the finger. X-ray imaging did not show any fractures, dislocations, or foreign bodies. A seven-day course of cephalexin was prescribed, with instructions to follow up with a hand surgeon three weeks later.

In the interim, he had persistent tenderness and swelling within the base of the ring finger, which began to radiate to the adjacent fingers and lower hand, and an inability to completely flex and extend the affected digits. He denied any fevers, chills, or night sweats. On physical examination, vital signs were normal and he was afebrile. His right upper extremity was grossly normal; however, the volar aspect of the proximal phalanx of the right ring finger had two small scars with a slight firmness of the scar. There was extensive swelling, specifically along the base of the ring finger. There was no palpable foreign body, although there was exquisite tenderness over the A1 pulleys of the right fourth and fifth fingers. He was able to make a full fist without difficulty, and the affected fingertips were neurovascularly intact. No bruising, cellulitis, erythema, or induration of the area was noted, and there was no lymphadenopathy within the right upper extremity.

**Figure 2:**
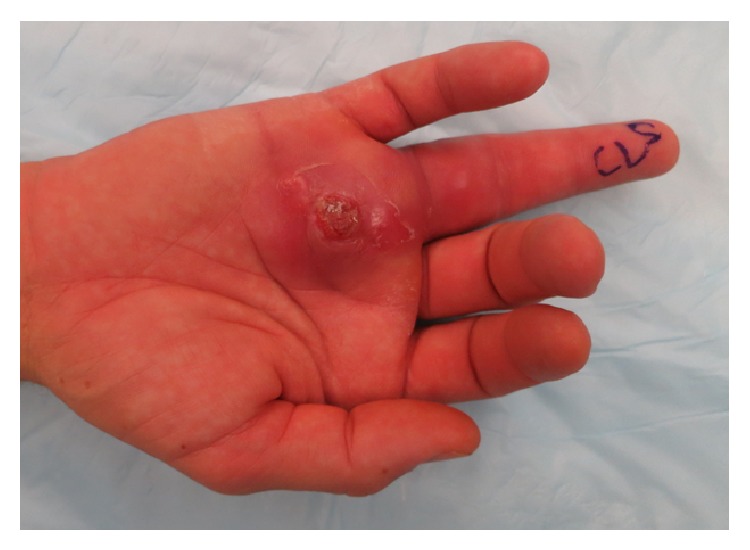
Tenosynovitis of the flexor digitorum superficialis (FDS) and flexor digitorum profundus (FDP) tendons. Clear nonpurulent fluid was noted around the tendons. A small puncture area was explored, and no foreign bodies were seen. Tissue samples were sent for pathology and routine and acid fast bacilli (AFB) cultures. Due to the high concern for a potential mycobacterial infection, clarithromycin, moxifloxacin, and rifabutin were started empirically. Three days later, he discontinued these medications due to gastrointestinal side effects from rifabutin without informing his physicians. Exudative cultures were negative; however, AFB cultures were positive, consistent with* Mycobacterium marinum*.

Surgical exploration of the flexor tendon sheath was performed, during which tenosynovitis was noted of the flexor digitorum superficialis (FDS) and flexor digitorum profundus (FDP) tendons (Figure 2). Clear nonpurulent fluid was noted around the tendons. A small puncture area was explored, and no foreign bodies were seen. Tissue samples were sent for pathology and routine acid fast bacilli (AFB) cultures. Due to the high concern for a potential mycobacterial infection, clarithromycin, moxifloxacin, and rifabutin were started empirically. Three days later, he discontinued these medications due to gastrointestinal side effects from rifabutin without informing his physicians. Exudative cultures were negative; however, early on cultures were positive for AFB, which were later confirmed to be* M. marinum*. Of note, tissue pathology showed fibroconnective tissue with extensive acute and chronic inflammation and Kinyoun stain was positive, consistent with atypical mycobacterial involvement.

**Figure 3:**
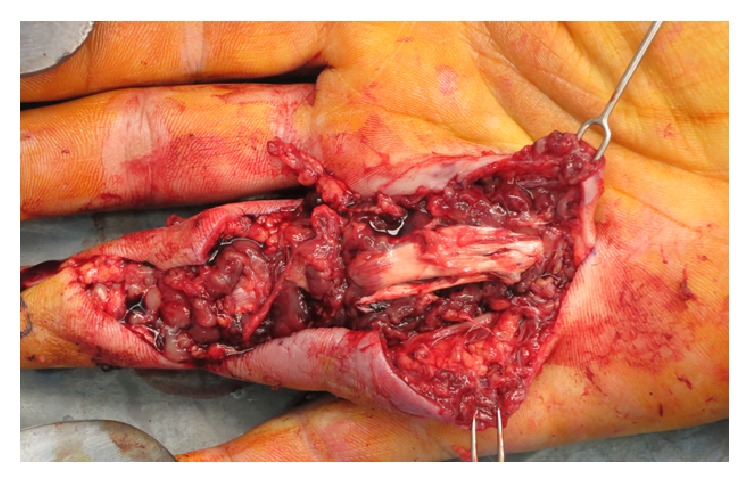
Debridement and radical synovectomy of his flexor tendon sheath secondary to necrotizing soft tissue infection. Erythema and induration surrounded the open wound, as well as nonviable wound edges that failed to bleed with manipulation, raising concern for a necrotizing soft tissue infection. He was immediately taken for surgical debridement and radical synovectomy of the right ring finger flexor tendon sheath, and tissue from the tendon sheath was sent for routine and mycobacterial cultures. Intravenous clarithromycin and moxifloxacin were initiated to empirically cover any potential superinfection. Twenty-four hours after admission, he was discharged to complete a six-month course of oral moxifloxacin and clarithromycin. AFB cultures were retuned positive, consistent with persistent* Mycobacterium marinum.*

At one-month postoperative follow-up, he had worsening pain and swelling, discharge, and wound discoloration. Vital signs were normal and he was afebrile. The wound had opened spontaneously and was draining a clear to “dishwater” colored fluid. Physical examination noted significant edema of the right ring finger and the distal palm over the A1 and A2 pulleys. Erythema and induration surrounded the open wound, as well as nonviable wound edges that failed to bleed with manipulation, raising concern for a necrotizing soft tissue infection. He was immediately taken for surgical debridement and radical synovectomy of the right ring finger flexor tendon sheath (Figure 3), and tissue from the tendon sheath was sent for routine and mycobacterial cultures. Intravenous clarithromycin and moxifloxacin were initiated along with ceftaroline to empirically cover potential superinfection. Twenty-four hours after admission, he was discharged to complete a six-month course of oral moxifloxacin and clarithromycin, in which he strictly adhered to this antimicrobial regimen, and close follow-up visits while on antibiotics were scheduled.

**Figure 4:**
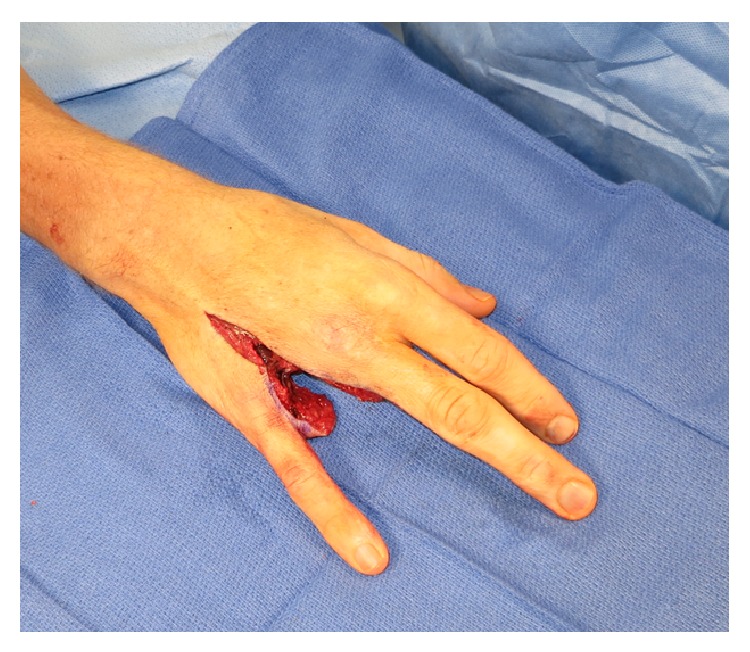
Persistent necrotizing soft tissue infection resulting in a ray amputation. Approximately one month following the second surgery, he presented to clinic with persistent swelling, erythema, induration, and dark necrotic appearing tissue along the base of the affected finger, consistent with persistent necrotizing soft tissue infection secondary to mycobacterial involvement. Ray amputation of the right ring finger was performed, and tissue pathology was consistent with granulomatous inflammation and necrosis, indicating a necrotizing soft tissue infection. Oral moxifloxacin and clarithromycin were continued to complete 6 months of therapy. During this time, he had marked clinical improvement and no evidence of persistent necrotizing soft tissue infection.

At the one-week after discharge follow-up visit, he had ongoing tenderness along the affected digit. Vital signs were normal and he was afebrile. Physical examination noted a closed incision with intact sutures. Erythema and induration were still present; however, no discharge was noted. There was moderate edema throughout his right ring finger, but the FDS and FDP tendons were intact with limited excursion. Approximately one month following the second surgery, he presented to clinic with persistent swelling, erythema, induration, and dark necrotic appearing tissue along the base of the affected finger, consistent with persistent necrotizing soft tissue infection. Of note, AFB cultures from the second surgery were again positive for persistent mycobacterial involvement. Ray amputation of the right ring finger was performed (Figure 4), and tissue pathology was consistent with granulomatous inflammation and necrosis, indicating a necrotizing soft tissue infection. Oral moxifloxacin and clarithromycin were continued to complete 6 months of therapy. During this time, he had marked clinical improvement and no evidence of persistent necrotizing soft tissue infection.

## 3. Conclusions

Due to recent public health efforts, cutaneous infections caused by* Mycobacterium marinum *that are associated with nonchlorinated swimming pools have decreased to less than 5% of the total cases [2]. We searched the English-language literature published until November 2014 in the PubMed database. Relevant studies were identified using the keyword combinations “necrotizing soft tissue infection,” “necrotizing fasciitis,” and “*Mycobacterium marinum.*” No lower publication data limit was set.

*Mycobacterium marinum* is a nontuberculosis mycobacterium that is a causative agent of human skin infections acquired through aquatic sources. Minor traumas, such as abrasions and lacerations, serve as the portal of entry for the organism. In recent decades, most cases of* M. marinum* have been reported after exposure to contaminated aquarium water or contact with fish and shellfish. Individuals at highest risk for exposure include fisherman and water sport athletes. We believe that our presenting case is unique in that most of the cases described in literature have responded well to either medical or surgical intervention; however, our case required multiple aggressive wound debridements, followed by an amputation of affected extremity secondary to a persistent necrotizing soft tissue infection caused by* M. marinum* that failed initial attempts of both medical and surgical intervention.

In most reported cases, the upper limbs and fingers account for a majority of infection sites [3, 4]. Clinical manifestations of superficial* M. marinum* infection typically include painless clusters of superficial nodules or papules. In about one-third of cases, lesions spread proximally along the lymphatic system in a* sporotrichoid* fashion, without associated lymphadenopathy [4, 5]. Most infections follow an indolent course, although disseminated cases have been reported in immunocompromised patients [6–8]. Deeper infections, including osteomyelitis and tenosynovitis, can cause significant morbidity if untreated [5, 7, 9].

In general, the average inoculation period for* M. marinum* is around 2–4 weeks; however, as seen with our case, a longer inoculation period can be seen. Other case reports have also described a prolonged incubation period [3, 4]. Failure to obtain an adequate history of aquatic exposure often occurs, since the preceding trauma is generally minor and unrecalled by the patient [4, 5]. Histology and bacteriology are confirmatory tests of infection; however, they are often difficult to use [5].

Treatment should be tailored to prevent progression to deeper infections and hasten recovery [1–3]. Optimal treatment usually involves appropriate drug therapy supplemented by surgical intervention for medical failure. Currently, there is no consensus on definitive treatment regimens due to paucity of controlled trials [3]. However, most isolates are resistant to isoniazid and pyrazinamide and produce *β*-lactamase [3, 10].

Several studies have shown effective eradication with a single antibiotic for uncomplicated cases, although most experts recommend 2-drug therapy [3, 10]. Clarithromycin alone, or in combination with ethambutol or rifampin, is preferred for superficial infections [2, 10]. Minocycline, doxycycline, or trimethoprim-sulfamethoxazole can be substituted for clarithromycin [10]. Duration of therapy ranges from 6 to 20 weeks, with some experts recommending continuation of treatment 1-2 months or longer, after resolution of skin lesions, as described in our case [3]. Deeper infections are best treated with combination of ethambutol and rifampin and surgical debridement [5].

While treatment of wound infections most commonly involves opening the incision, a necrotizing soft tissue infection, the most serious consequence of any wound infection, may require more aggressive surgical intervention [4, 11]. Wound infections resulting in a necrotizing soft tissue infection caused by* M. marinum* often present with a papule, nodule, or ulcer at the site of their trauma, along with a pertinent history of exposure to nonchlorinated water 2–4 weeks earlier [7]. With unrecognized or untreated cases, these infections often ascend up the finger or hand or spread to involve a local joint or tendon [7]. Over a short period of weeks to months, these localized cutaneous infections can spread to soft tissues, ultimately resulting in tissue necrosis [8]. Pertinent signs and symptoms typically include localized pain, edema, and induration surrounding an infected wound, with or without a nonpurulent exudate [11]. With the exception of immunocompromised hosts, fever, lymphadenopathy, and systemic infection are uncommon. Early intervention is prudent to prevent further extension of infection and, therefore, further tissue necrosis.

In our presenting case, prompt surgical wound debridement was performed, secondary to persistent erythema, swelling, a clear to dishwater colored fluid, and evidence of nonviable necrotic wound edges. Although closely followed up in our clinic with careful inspection and monitored antibiotic therapy, he did not improve. Continued debridement, with all efforts to salvage the affected finger, was undertaken; however, due to persistent* Mycobacterium marinum* involvement and evidence of nonviable necrotic wound edges, a ray amputation was performed to prevent further compromise of the entire right hand and adjacent fingers. Thus, aggressive surgical debridement in combination with judicious antimicrobial therapy is needed for necrotizing infections [12].

A high index of suspicion is needed in those with cutaneous lesions and a history of aquatic encounter. Since the organism can take several weeks to culture, patients should be preemptively treated with appropriate antibiotics to hasten the disease course. Misdiagnosis or delay in treatment may result in worsening of clinical symptoms and progression to deeper infection. Dual therapy is the mainstay of treatment, although selected cases may require surgical intervention.
